# MOR23 deficiency exacerbates hepatic steatosis in mice

**DOI:** 10.1096/fj.202401468RR

**Published:** 2024-10-17

**Authors:** Wesuk Kang, Suhjin Yang, Jiyun Roh, Dabin Choi, Han‐Woong Lee, Jae Hoon Lee, Taesun Park

**Affiliations:** ^1^ Department of Food and Nutrition, BK21 FOUR Yonsei University Seoul Republic of Korea; ^2^ Department of Biochemistry, College of Life Science and Biotechnology Yonsei University, Gemcro, Inc. Seoul Republic of Korea

**Keywords:** cedrene, hepatic steatosis, knockout mice, mouse olfactory receptor 23, olfactory receptor

## Abstract

Hepatic steatosis, a common liver disorder, can progress to severe conditions such as nonalcoholic steatohepatitis and cirrhosis. While olfactory receptors are primarily known for detecting odorants, emerging evidence suggests that they also influence liver lipid metabolism. This study generated a mouse model with a specific knockout of olfactory receptor 23 (MOR23) to investigate its role in hepatic steatosis. MOR23 knockout mice on a normal diet showed a slight increase in liver weight compared to wild‐type (WT) mice. When fed a high‐fat diet (HFD), these knockout mice exhibited accelerated hepatic steatosis, indicated by increased liver weight and hepatic triglyceride levels. Our findings suggest that the cyclic adenosine monophosphate/protein kinase A/AMP‐activated protein kinase pathway is involved in the role of MOR23, leading to the upregulation of peroxisome proliferator‐activated receptor α, peroxisome proliferator‐activated receptor‐γ coactivator 1‐α, and their target β‐oxidation genes in the liver. MOR23 also appeared to regulate lipogenesis and free fatty acid uptake in HFD‐fed mice, potentially by influencing sterol regulatory element‐binding protein 1 activity. Notably, administering a potential MOR23 ligand, cedrene, attenuated hepatic steatosis in WT mice, but these effects were largely nullified in MOR23 knockout mice. These findings provide valuable insights into the in vivo role of MOR23 in hepatic steatosis development.

## Graphical abstract

MOR23 deficiency accelerates hepatic steatosis through the cAMP/PKA/AMPK pathway, increasing lipogenesis and free fatty acid uptake while decreasing β‐oxidation in mice fed a high‐fat diet. Cedrene administration mitigates these effects in wild‐type mice but not in MOR23 knockout mice, highlighting the therapeutic potential of MOR23 activation in managing hepatic steatosis and its associated metabolic disturbances.

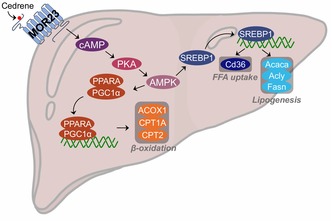

**Abbreviations**
Acaca: acetyl‐Coenzyme A carboxylase alpha
Acadm: acyl‐Coenzyme A dehydrogenase; medium chain
Acly: ATP citrate lyase
Acox1: acyl‐CoA oxidase 1
ALT: alanine aminotransferase
AMPK: AMP‐activated protein kinase
AST: aspartate aminotransferase
cAMP: cyclic adenosine monophosphate
Cd36: cluster of differentiation 36
Cpt1a: carnitine palmitoyltransferase 1A
Fasn: fatty acid synthase
FER: feed efficiency ratio
FFA: free fatty acid
GAPDH: glyceraldehyde‐3‐phosphate dehydrogenase
GPCR: G‐protein‐coupled receptor
H&E: hematoxylin and eosin
HFD: high‐fat diet
KO: knockout
MOR23: mouse olfactory receptor 23
NAFLD: nonalcoholic fatty liver disease
ND: normal diet
OR10J5: olfactory receptor family 10 subfamily J member 5
PGC1α: peroxisome proliferator‐activated receptor‐γ coactivator 1‐α
PKA: protein kinase A
Pnpla2: patatin‐like phospholipase domain‐containing protein 2
PPARα: peroxisome proliferator‐activated receptor α
Scd1: stearoyl‐coenzyme A desaturase 1
SEM: standard error of the mean
SREBP1: sterol regulatory element‐binding protein 1
TG: triglycerides
WT: wild type

## 1 INTRODUCTION

Nonalcoholic fatty liver disease (NAFLD) is a prevalent disorder characterized by the accumulation of excess fat in the liver. This condition is a significant risk factor for the development of severe liver conditions, such as nonalcoholic steatohepatitis and cirrhosis, and is frequently associated with obesity.^1^, ^2^, ^3^ The pathogenesis of NAFLD is complex and involves several molecular pathways, including the uptake of free fatty acids (FFAs) and the regulation of lipogenesis and β‐oxidation.^4^, ^5^, ^6^ Recent studies have identified several potential molecular targets for the treatment of NAFLD, including specific receptors involved in the regulation of FFA uptake.^7^, ^8^, ^9^ Despite significant progress in our understanding of the mechanisms underlying NAFLD, the development of effective therapies remains a major challenge. One reason for this is that these mechanisms are interconnected rather than independent and must be considered as a whole to effectively target them.

Olfactory receptors are a type of G‐protein‐coupled receptor that plays a role in the detection and identification of odorants in the nose. However, recent research has shown that these receptors can also be expressed and function outside the olfactory system.^10^, ^11^ When the olfactory receptor binds to specific odorants, it initiates a process involving the production of cyclic adenosine monophosphate (cAMP) and activation of protein kinase A (PKA).^12^, ^13^ PKA activation leads to the phosphorylation of various protein kinases, including AMP‐activated protein kinase (AMPK). AMPK is a key metabolic enzyme that regulates energy balance in the body, particularly overall lipid metabolism, including lipogenesis and β‐oxidation in the liver.^14^, ^15^, ^16^ For example, olfr43, an isotype of the olfactory receptor expressed in mouse hepatocytes, has been reported to function as a nonredundant receptor that regulates cAMP signaling and modulates liver steatosis in response to its ligand carvone.^17^ Furthermore, olfr544 is highly expressed in the liver and promotes fatty acid oxidation by its ligand, azelaic acid.^18^ This suggests that targeting the olfactory receptor may be a promising therapeutic strategy for the treatment of fatty liver disease.

The previous studies have suggested that the mouse olfactory receptor 23 (MOR23, olfr16) and its human orthologue, olfactory receptor family 10 subfamily J member 5 (OR10J5), are involved in angiogenesis, regulation of sperm motility, and proper skeletal muscle regeneration at the in vitro level.^19^, ^20^, ^21^ Recently, our research showed that the knockdown of OR10J5 using siRNA in human hepatocytes led to increased lipid accumulation.^22^ While the ligands for most mammalian olfactory receptors are not yet identified, we discovered that MOR23 and OR10J5 recognize α‐cedrene, a sesquiterpene component of cedarwood oil, as a potential agonist. Furthermore, the activation of OR10J5 by this ligand significantly reduces fatty‐acid‐induced lipid accumulation in human hepatocytes.^22^ However, the liver, being the largest internal organ and composed of various cell types, plays a complex and crucial role in multiple metabolic processes. Hence, the contribution of specific receptors to hepatic steatosis cannot be fully represented in an in vitro setting using hepatocytes alone and, therefore, should be systematically assessed. In this study, we have created a knockout (KO) mouse model lacking MOR23 to investigate its role in hepatic steatosis and explore the olfactory‐receptor‐mediated signaling pathway in this mouse model.

## 2 MATERIALS AND METHODS

### 2.1 Animal experiments

CRISPR/Cas9‐mediated whole‐body MOR23 knockout (KO) mice were generated by GEMCRO Inc. (Seoul, Korea) using Toolgen's technology on a C57BL/6 strain background. The mice were housed in a controlled environment with a 12‐h light/dark cycle, temperature maintained at 22 ± 1°C, and a relative humidity of 50 ± 5%. Both male and female KO mice, along with their sex‐matched wild‐type (WT) littermates, were randomly assigned at 8 weeks of age to either the normal diet (ND) group or the high‐fat diet (HFD) group, with or without 1% α‐cedrene (w/w). The ND group was fed a purified diet based on the AIN‐76 rodent diet composition. The HFD group had an identical diet to the ND group, except for the addition of 200 g fat/kg (comprising 170‐g lard and 30‐g corn oil) and 1% cholesterol (Table S1). This diet composition ensures that 40% of the total energy provided by the diet comes from fat. Throughout the 10‐week study period, the mice had ad libitum access to water and their designated experimental diets. The body weights of the mice were recorded weekly, and food intake was measured daily over a 10‐week period. Each day, a preweighed amount of food was provided to each cage, and the remaining food was collected and weighed after 24 h. Daily food intake was calculated as the difference between the initial and remaining food weights. At the end of the experiment, after a fasting period of 6 h, the mice were anesthetized using Avertin (Sigma‐Aldrich, Burlington, MA, USA). Tissues were then dissected, rinsed, weighed and stored at −80°C for subsequent analyses. All animal experiments were approved by the Institutional Animal Care and Use Committee (Grant Number: IACUC‐A‐202305‐1680‐01) of the Yonsei Laboratory Animal Research Center.

### 2.2 Biochemical analysis

Trunk blood was collected from the portal vein of the mice and transferred into ethylenediaminetetraacetic acid (EDTA)‐coated tubes. The plasma was separated by centrifugation at 2000 *g* for 15 min at 4°C. To extract hepatic lipids, the method described by Folch et al.^23^ was used, employing a chloroform–methanol mixture (2:1 v/v) and re‐dissolving the lipids in ethanol. The levels of triglycerides (TG) and FFA were quantified using enzymatic colorimetric assays with commercial kits from Biomax (Seoul, Korea). Glycerol concentration was determined using the PicoSens Free Glycerol Assay Kit (Biomax). Plasma concentrations of aspartate aminotransferase (AST) and alanine aminotransferase (ALT) were evaluated by enzymatic colorimetric tests using kits from Asan Pharmaceutical (Seoul, Korea). Absorbance readings were taken at 450 nm using an Infinite M200 microplate reader (Tecan, Männedorf, Switzerland). All procedures were conducted following the manufacturer's guidelines.

### 2.3 Histological analysis

The harvested liver tissues were fixed in 4% paraformaldehyde, embedded in paraffin blocks, and sectioned to a thickness of 5 μm. Subsequently, the sections were deparaffinized using xylene and rehydrated using a series of ethanol solutions with decreasing concentrations. The representative sections were then stained with hematoxylin and eosin and observed using a Nikon Eclipse TS2 microscope equipped with a DMX1200 camera (Nikon, Tokyo, Japan). The area of lipid droplets in the stained liver sections was quantitatively assessed using ImageJ software (National Institutes of Health, Bethesda, MD, USA).

### 2.4 Protein extraction and Western blot analysis

The tissues were homogenized in ice‐cold RIPA buffer (Thermo Fisher) containing protease and phosphatase inhibitor cocktails (Sigma‐Aldrich). The total protein concentrations were determined using a BCA Protein Assay Kit (Takara, Tokyo, Japan). Equal amounts of these protein extracts were separated by SDS‐PAGE using a 12% gel and transferred to a nitrocellulose membrane (Bio‐Rad, Hercules, CA, USA). The membrane was then blocked with 5% bovine serum albumin (BSA; MP Biomedicals, Santa Ana, CA, USA) in Tris‐buffered saline containing 0.1% Tween 20 (TBS‐T) for 1 h and probed with primary antibodies (Cell Signaling Technology, Boston, MA, USA) against p‐AMPK (1:1000), AMPK (1:1000), PKA (1:1000), sterol regulatory element‐binding protein 1 (SREBP1, 1:1000), cluster of differentiation 36 (Cd36; 1:1000) and glyceraldehyde‐3‐phosphate dehydrogenase (GAPDH, 1:5000) for 16 h at 4°C. After washing the membrane three times with TBS‐T for 5 min each, horseradish peroxidase‐conjugated secondary antibodies (Sigma‐Aldrich; 1:10 000) were added and incubated for 1 h at 20°C. Following another three washes with TBS‐T for 10 min each, the blot was developed using a chemiluminescent substrate (WestGlow PICO PLUS; Biomax, Seoul, Korea) and visualized using a ChemiDoc imaging device (ATTO, Tokyo, Japan). The band intensities were analyzed using Image J software 1.54 j (Rasband, W.S., ImageJ, U.S. National Institutes of Health, Bethesda, Maryland, USA, https://imagej.net/ij/, 1997–2018).

### 2.5 RNA extraction and real‐time PCR (RT‐PCR)

Total RNA was extracted from liver tissues using TRIzol reagent (Invitrogen, Carlsbad, CA, USA), followed by chloroform extraction and isopropanol precipitation (Sigma‐Aldrich). The RNA was eluted in nuclease‐free water, quantified using NanoDrop 2000 (Thermo Fisher), and reverse transcribed with the PrimeScript RT Reagent Kit with gDNA Eraser (Takara, Tokyo, Japan) according to the manufacturer's instructions. RT‐PCR was performed in a final volume of 20 μL, which included 10 μL of TB Green Premix (Takara), primers at a concentration of 0.4 μM each, and 20 ng of complementary DNA. The thermal cycling conditions were as follows: Initial denaturation at 95°C for 30 s, followed by 35 cycles of denaturation at 95°C for 5 s, and annealing and extension at 58°C for 40 s. The results were calculated using the 2^−ΔΔCt^ method, with gene expression normalized to GAPDH. Primer sequences are presented in Table S2.

### 2.6 PKA activity measurements

PKA activity in liver tissues from WT and MOR23 KO mice was assessed using a commercial PKA activity kit (Thermo Fisher). The samples were added to a plate containing immobilized PKA substrate, treated with ATP, and incubated at 20°C for 2 h. Subsequently, the plate was incubated with the phospho‐PKA substrate antibody for 60 min. After washing, goat anti‐rabbit IgG conjugated to horseradish peroxidase was added. Following another wash, tetramethylbenzidine substrate was added and incubated for an additional 30 min at 20°C. The absorbance was evaluated at 450 nm using a microplate reader (Tecan).

### 2.7 Glycerol release assay

White adipose tissues of the epididymis were excised from euthanized WT and KO mice and immediately placed in cold phosphate‐buffered saline. Freshly isolated adipose tissue was minced into small pieces and incubated in Krebs‐Ringer bicarbonate buffer supplemented with 3.5% BSA and 5 mM glucose at 37°C with gentle agitation. After a 2‐h incubation, the supernatants were collected by centrifugation at 1000 *g* for 10 min. The glycerol content in the supernatants was measured using a glycerol assay kit (Biomax) according to the manufacturer's instructions. Glycerol release was normalized to tissue weight to account for the variability in tissue size among the samples.

### 2.8 Statistical analysis

The data are presented as the mean ± standard error of the mean (SEM). Statistical differences between the two groups were evaluated using an unpaired two‐tailed Student's *t*‐test. When statistical differences among four groups were analyzed, two‐way ANOVA was used to test for significant main effects (genotype, G; diet, D) and a G × D interaction. When significant main effects or interactions were observed, the Tukey's post‐hoc test was used to determine differences among the groups. All statistical analyses were performed using GraphPad Prism 9.4.1 (GraphPad Software, San Diego, CA, USA). The significance levels were set at *p < .05, **p < .01, and ***p < .001.

## 3 RESULTS

### 3.1 No phenotypic differences were observed between wild‐type (WT) and MOR23 KO mice fed a normal diet (ND)

**FIGURE 1:**
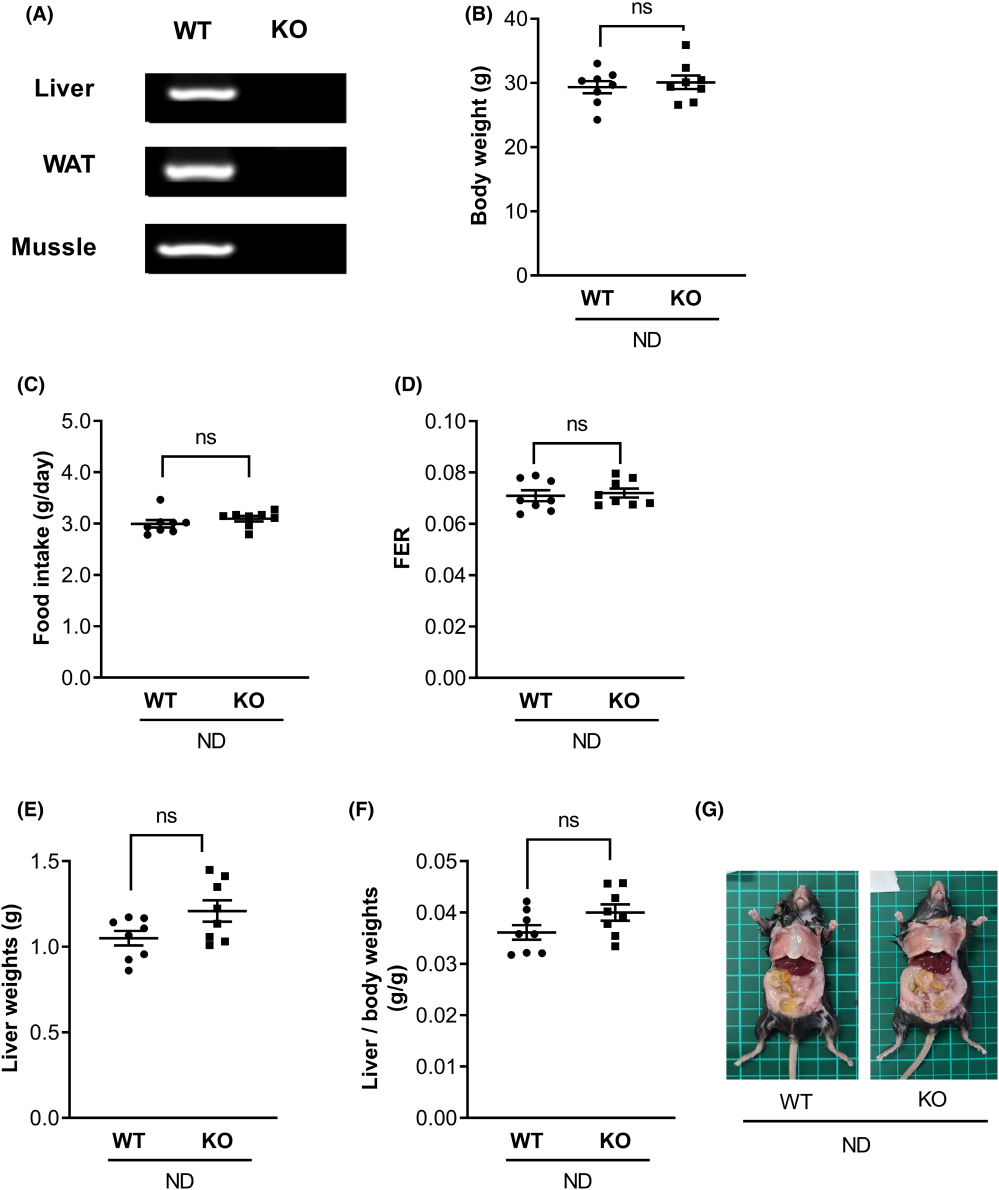
No phenotypic differences were observed between wild‐type (WT) and MOR23 knockout (KO) mice fed a normal diet (ND). (A) Validation of MOR23 knockout by PCR analysis in the liver, adipose, and muscle tissues of WT and MOR23 knockout (KO) mice. (B–D) Examination of body weight, food intake, and feed efficiency ratio (FER) in WT and MOR23 KO male mice maintained on an ND for 10 weeks. (E–G) Assessment of liver weight, liver weight relative to body weight, and representative images of dissected livers from WT and MOR23 KO mice. Data are represented as the mean ± standard error of the mean (SEM). *n* = 8 mice per group. ns, not significant.

MOR23 KO mice with a C57BL/6 background were generated by deleting MOR23 exon 1 using CRISPR/Cas9. As expected, PCR analyses confirmed the absence of MOR23 expression in key tissues, including the liver, adipose tissues, and muscle tissues (Figure 1A).

The objective of this study was to examine phenotypic differences between wild‐type (WT) and MOR23 KO mice. Both groups of male mice were given an ND for a period of 10 weeks, during which their body weight, food consumption, and feed efficiency ratio (FER) were assessed. Our findings revealed no significant changes in body weight, food intake, and FER in MOR23 KO mice on ND compared to their WT counterparts (Figure 1B–D).

Inspired by a previous study that reported knockdown of OR10J5 (a human ortholog of MOR23) leading to increased lipid accumulation in hepatocytes, we aimed to investigate the potential role of MOR23 in hepatic steatosis. We focused on phenotypic alterations in the liver of MOR23‐deficient mice and evaluated liver weight normalized to body weight. Although no significant differences in liver weight or morphology were observed, we detected a tendency toward a higher liver weight‐to‐body weight ratio in MOR23 KO mice compared to their WT littermates (Figure 1E–G).

Similarly, female mice were maintained on a normal diet for 10 weeks to explore potential phenotypic differences. Consistent with the data from male mice, there were no significant changes in body weight between the female MOR23 KO and WT groups (Figure S1A). However, an increase in the liver weight‐to‐body weight ratio was observed in the KO group compared to the WT group, reflecting a similar trend seen in the male mice. This suggests a potential involvement of MOR23 in liver size regulation across both sexes (Figure S1B).

### 3.2 The deficiency of MOR23 leads to the uptake of FFA from plasma into the liver of mice under ND

**FIGURE 2:**
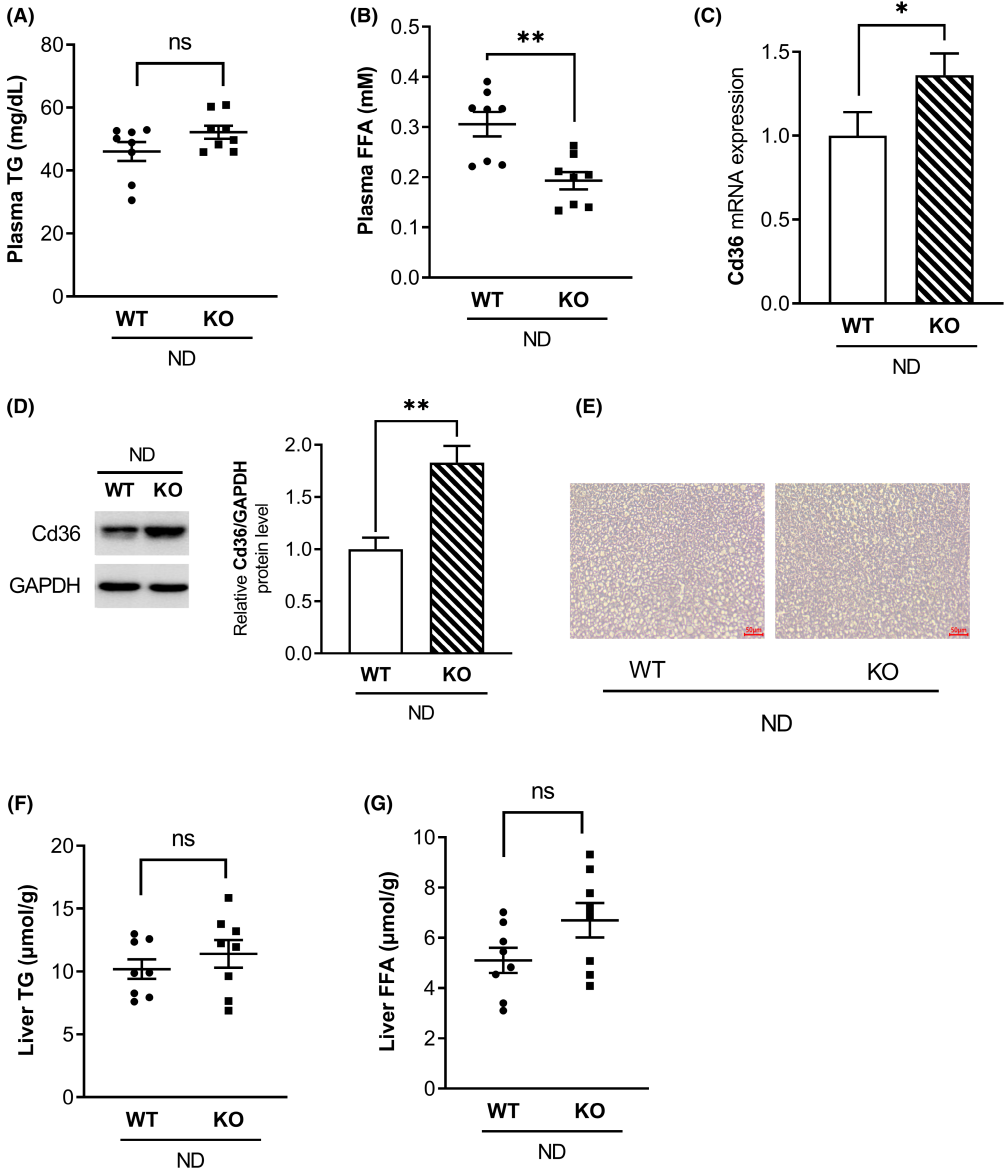
The deficiency of MOR23 leads to the uptake of free fatty acids (FFA) from plasma into the liver of mice under an ND. (A, B) Plasma triglyceride (TG) and FFA levels in WT and MOR23 KO mice fed an ND. (C) mRNA expression of cluster of differentiation 36 (Cd36) in WT and MOR23 KO mice. (D) Protein expression of Cd36 in the liver of MOR23 KO and WT mice. (E) Hematoxylin and eosin (H&E) staining of liver sections from WT and MOR23 KO mice fed an ND (scale bar = 50 μm). (F, G) Liver TG and FFA contents in WT and MOR23 KO mice. Data are presented as means ± SEM. For (A), (B), (F), and (G), *n* = 8 mice per group. For (C) and (D), samples from each group of eight mice were pooled into three biological replicates, with each replicate consisting of three, three, and two mice, respectively. Significant differences between groups are indicated by asterisks; *p < .05; **p < .01. ns, not significant.

To explore the potential impact of MOR23 deficiency on lipid profiles, we examined biochemical markers in the blood of both WT and MOR23 KO mice fed an ND. Our first analysis focused on plasma TG and FFA levels to assess the influence of MOR23 deficiency. The results showed no significant difference in plasma TG levels between the MOR23 KO and WT groups (Figure 2A). However, the MOR23 KO group exhibited significantly lower plasma FFA levels compared to the WT group on an ND (Figure 2B).

To account for the reduced plasma FFA levels in MOR23 KO mice, we investigated two possible explanations: (1) increased FFA uptake in the liver or (2) decreased lipolysis in adipose tissue. To explore the first possibility, we measured the mRNA expression of cluster of differentiation 36 (Cd36), a marker for hepatic fatty acid uptake. Interestingly, Cd36 mRNA expression was significantly higher in MOR23 KO mice than in WT controls (Figure 2C). To determine whether the increased Cd36 mRNA expression in MOR23 KO mice translated to higher protein levels, we measured hepatic Cd36 protein expression using Western blot analysis. Consistent with the mRNA data, Cd36 protein levels were significantly elevated in the livers of MOR23 KO mice compared to WT controls (Figure 2D). In contrast, there were no significant differences in plasma glycerol levels, glycerol release from adipose tissue, or the expression of patatin‐like phospholipase domain‐containing protein 2 (Pnpla2), a representative lipolysis gene, between the two groups (Figure S1A–C). Based on these findings, we concluded that the decrease in plasma FFA levels in the MOR23 KO group, compared to the WT controls, was likely due to increased FFA uptake in the liver.

Having established this, we then hypothesized that MOR23 KO could lead to significant hepatic steatosis. To investigate this, we assessed lipid accumulation in liver sections from WT and MOR23 KO mice through hematoxylin and eosin (H&E) staining. As shown in Figure 2E, there were no noticeable differences in liver histology between the two groups fed an ND. Analysis of TG and FFA contents also revealed no significant difference in TG levels between MOR23 KO mice and WT controls. However, there was a slight increase in plasma FFA levels in the MOR23 KO group, although this increase was not statistically significant (Figure 2F,G).

### 3.3 A significant increase in liver weight was observed in MOR23 KO mice fed an HFD

**FIGURE 3:**
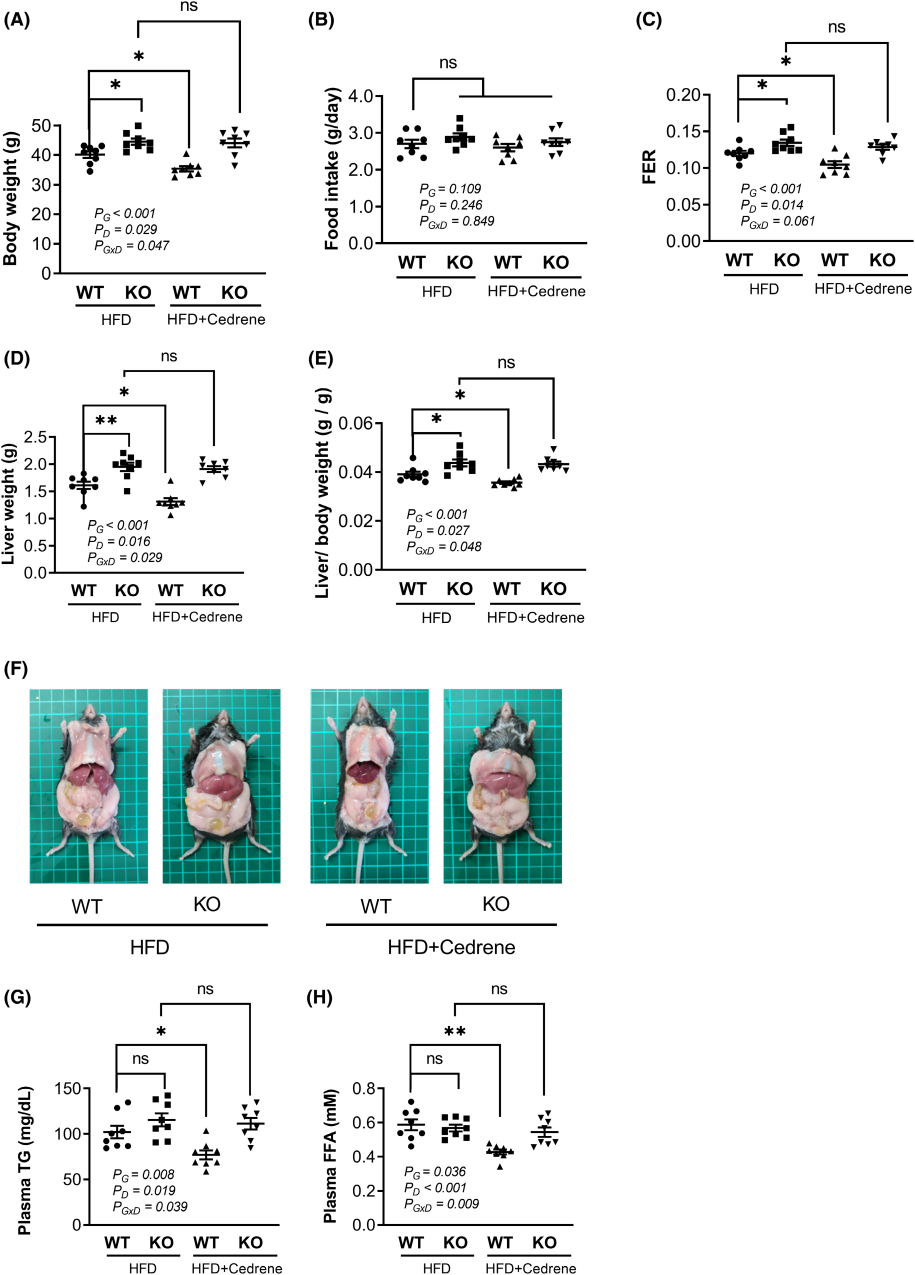
A significant increase in liver weight was observed in MOR23 KO mice fed a high‐fat diet (HFD). (A–C) Body weight and food consumption in WT and MOR23 KO male mice fed an HFD with or without cedrene treatment. (D, E) Liver weight measurements in WT and MOR23 KO mice fed an HFD with or without cedrene treatment. (F) Representative images of dissected organs, illustrating the visible patterns of liver changes. (G, H) Plasma TG and FFA levels in WT and MOR23 KO mice fed an HFD with or without cedrene treatment. The data are presented as the mean ± SEM (*n* = 8 per group). A two‐way analysis of variance (ANOVA) was performed to assess the main effects (G, Genotype; D, Diet) and their interaction (G × D), as indicated within the graphs. Tukey's multiple‐comparison test was used to compare individual means, and statistical significance between groups is indicated by asterisks; ns, not significant; *p < .05; **p < .01. ns, not significant.

To conduct a more comprehensive examination of the involvement of MOR23 in the development of hepatic steatosis, we employed an HFD to induce hepatic lipid accumulation. Additionally, we supplemented the HFD with cedrene, an MOR23 agonist. After a 10‐week treatment period, we observed a significant increase in body weight in MOR23 KO mice compared to WT mice, despite similar food consumption. Notably, the administration of cedrene resulted in a considerable reduction in body weight in HFD‐fed WT mice, but this effect was nullified in MOR23 KO mice (Figure 3A–C).

Motivated by these findings, we sought to determine whether these weight changes were also reflected in the liver. We analyzed liver weight in the same experimental groups and discovered that the liver weight of MOR23 KO mice on the HFD significantly exceeded that of WT mice, surpassing the overall gain in body weight. Furthermore, the activation of MOR23 with cedrene led to a significant decrease in liver weight in WT mice. However, this effect was abolished in MOR23 KO mice (Figure 3D,E). Similar trends were also observed in the dissected organ images (Figure 3F).

In addition to changes in body and liver weight, we examined the effects of cedrene on plasma TG and FFA levels to further explore MOR23's role in hepatic steatosis. As shown in Figure 3G, TG levels exhibited a tendency to increase in MOR23 KO mice compared to WT mice under HFD conditions; however, this change was not statistically significant. For FFA levels (Figure 3H), knockout of MOR23 did not result in any noticeable change. Cedrene administration had a reducing effect on both plasma TG and FFA levels in WT mice, highlighting its potential role in mitigating lipid accumulation. Collectively, these results indicate that MOR23 plays a role in regulating overall weight gain, particularly within the liver, when exposed to an HFD, while causing minimal changes in the overall blood lipid profile.

To further explore the role of MOR23 in body and liver weight regulation concerning sex differences, we subjected female mice to an HFD with cedrene supplementation for 10 weeks. Consistent with the findings in males, MOR23 KO females showed a significant increase in body weight compared to WT mice and an elevated liver weight‐to‐body weight ratio. Cedrene reduced liver size in HFD‐fed WT females; however, this effect was absent in MOR23‐deficient mice (Figure S3A,B). These results suggest that MOR23 influences weight gain and liver size under HFD conditions, regardless of sex.

### 3.4 MOR23 regulates hepatic steatosis in a mouse model fed an HFD

**FIGURE 4:**
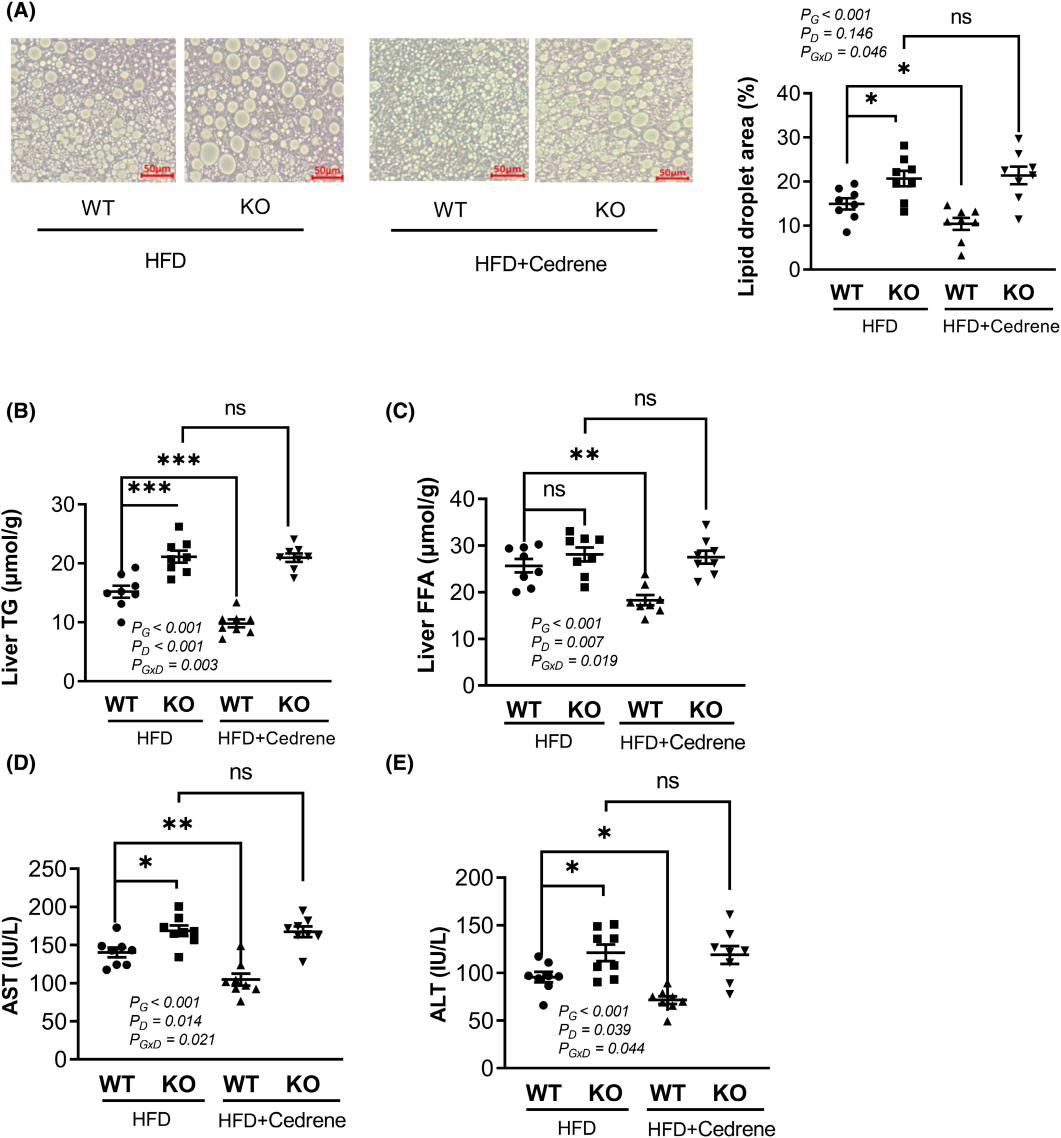
MOR23 regulates hepatic steatosis in a mouse model fed an HFD. (A) Histological examination of hepatic lipid accumulation using H&E staining in MOR23 KO and WT mice fed an HFD and cedrene (scale bar = 50 μm). The lipid droplet area was measured using ImageJ. (B, C) Biochemical analysis of hepatic TG and FFA levels in MOR23 KO and WT male mice fed an HFD and cedrene. (D, E) Plasma levels of aspartate aminotransferase (AST) and alanine aminotransferase (ALT) as markers of liver damage in MOR23 KO and WT mice subjected to HFD, with and without cedrene treatment. The data are presented as the mean ± SEM (*n* = 8 per group). A two‐way ANOVA was performed to assess the main effects (G, Genotype; D, Diet) and their interaction (G × D), as indicated within the graphs. Tukey's multiple‐comparison test was used to compare individual means, and statistical significance between groups is indicated by asterisks; *p < .05; **p < .01; ***p < .001; ns, not significant.

To further investigate the cause of the increase in liver weight, we quantified hepatic fat content by using H&E staining on liver tissue from both KO and WT mouse groups. Our findings revealed that HFD intake led to a significant increase in hepatic lipid droplets in KO mice compared to WT mice. When WT mice were fed HFD and cedrene, a decrease in lipid droplets was observed compared to WT mice fed only HFD. However, this cedrene‐induced reduction in hepatic lipid droplets was not observed in MOR23 KO mice (Figure 4A).

We further examined the biochemical markers of hepatic lipids, including TG and FFA in the livers of the same groups. Our results demonstrated that TG levels in the liver were significantly higher in MOR23 KO mice than in WT mice. Additionally, we found that TG levels were significantly decreased when WT mice were supplemented with HFD and cedrene, but this effect was not observed in KO mice (Figure 4B), which is consistent with the H&E staining results. In contrast, no significant difference was observed in liver FFA levels between WT and KO mice. Coadministration of the MOR23 ligand with HFD in WT mice resulted in a significant reduction in FFA levels compared to HFD alone, an effect that was not observed in KO mice (Figure 4C).

To assess the impact of MOR23 on hepatic lipid metabolism in female mice, we measured TG and FFA levels in the liver. The results showed a trend similar to those observed in males, though less significant. Specifically, TG levels were elevated in MOR23 KO females compared to WT. Cedrene treatment in HFD‐fed WT mice reduced TG levels, an effect not seen in KO females, indicating its dependence on functional MOR23 (Figure S4A). FFA content did not differ significantly between WT and KO females, but cedrene modestly reduced FFA in WT females (Figure S4B). Thus, MOR23 appears to have a similar influence on hepatic lipid accumulation in both sexes. After confirming that key markers, such as body and liver weight, as well as lipid profiles in the liver, exhibited similar patterns of hepatic steatosis in both sexes following MOR23 knockout and ligand administration, we chose to focus on a more detailed analysis using male mice as the representative model.

To determine whether the observed hepatic lipid accumulation in MOR23 KO mice ultimately led to liver damage, we measured the plasma levels of AST and ALT as markers of liver damage. Our results showed that the levels of both enzymes were significantly increased in MOR23 KO mice. In addition, coadministration of cedrene with HFD resulted in a significant reduction in AST and ALT levels compared to HFD alone. Notably, the effects of cedrene on reducing these enzyme levels were absent in MOR23 KO mice (Figure 4D,E). These results indicate that MOR23 plays a crucial role in regulating hepatic steatosis and liver damage in a mouse model fed an HFD.

### 3.5 MOR23 regulates the cAMP/PKA/AMPK pathway in the livers of mice fed an HFD

**FIGURE 5:**
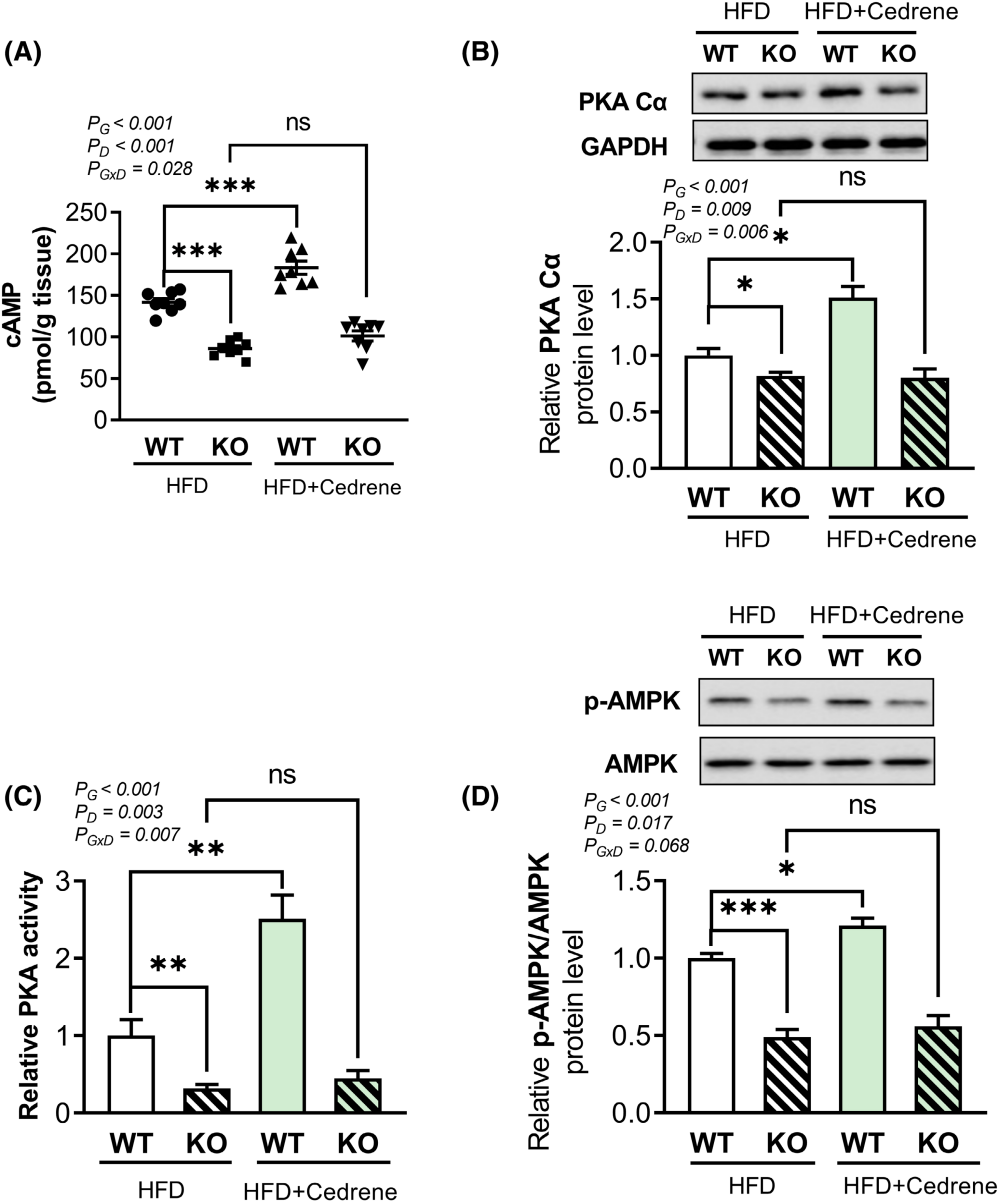
MOR23 regulates the cAMP/PKA/AMPK pathway in the livers of mice fed an HFD. (A) Measurement of cAMP levels in liver tissues from WT and MOR23 KO mice fed an HFD, with or without cedrene treatment, using enzyme‐linked immunosorbent assay (ELISA). (B) Quantification of protein kinase Cα (PKA Cα) levels in liver tissues of WT and MOR23 KO mice on an HFD, with or without cedrene treatment, using western blot. (C) Comparison of PKA activity in WT and MOR23 KO mice fed an HFD, with or without cedrene treatment. (D) Determination of phosphorylated AMP‐activated protein kinase (p‐AMPK) levels in liver tissues from WT and MOR23 KO mice on an HFD, with or without cedrene treatment, using western blot. Data are presented as mean ± SEM. For (A), *n* = 8 mice per group. For (B), (C), and (D), samples from each group of eight mice were pooled into three biological replicates, with each replicate consisting of three, three, and two mice, respectively. A two‐way ANOVA was performed to assess the main effects (G, Genotype; D, Diet) and their interaction (G × D), as indicated within the graphs. Tukey's multiple‐comparison test was used to compare individual means, and statistical significance between groups is indicated by asterisks; *p < .05; **p < .01; ***p < .001; ns, not significant.

In this study, we investigated the role of MOR23 in the regulation of liver steatosis and its underlying mechanisms. Specifically, we focused on the potential involvement of the cAMP/PKA pathway and AMPK in this regulation. We measured cAMP levels in the liver tissues of WT and MOR23 KO mice. We found that cAMP levels were significantly higher in WT mice fed an HFD and treated with the MOR23 ligand cedrene compared to WT mice fed an HFD alone. However, this effect was not observed in KO mice (Figure 5A). Furthermore, we assessed the levels of PKA Cα, a downstream target of cAMP, in response to MOR23 KO using a western blot assay. Our data revealed a significant decrease in PKA Cα levels in MOR23 KO mice compared to WT mice (Figure 5B). To evaluate the functional consequences of these changes, we measured PKA activity and observed a substantial reduction in PKA activity in KO mice compared to WT mice. Additionally, treatment with cedrene markedly increased PKA activity in WT mice by more than two‐fold, but had no effect in KO mice (Figure 5C).

We also examined the phosphorylation levels of AMPK and found that phosphorylated AMPK (p‐AMPK) was significantly reduced in MOR23 KO mice compared to WT mice, similar to the results obtained for cAMP and PKA. Moreover, mice fed an HFD and the ligand exhibited a significant increase in AMPK phosphorylation compared to mice fed an HFD alone, but this increase was not observed in MOR23 KO mice under the same conditions (Figure 5D). Overall, our findings suggest that the cAMP/PKA/AMPK signaling pathway is involved in the MOR23‐mediated regulation of liver steatosis.

### 3.6 MOR23 regulates PPARα/PGC1α pathway and β‐oxidation genes in HFD‐fed mice

**FIGURE 6:**
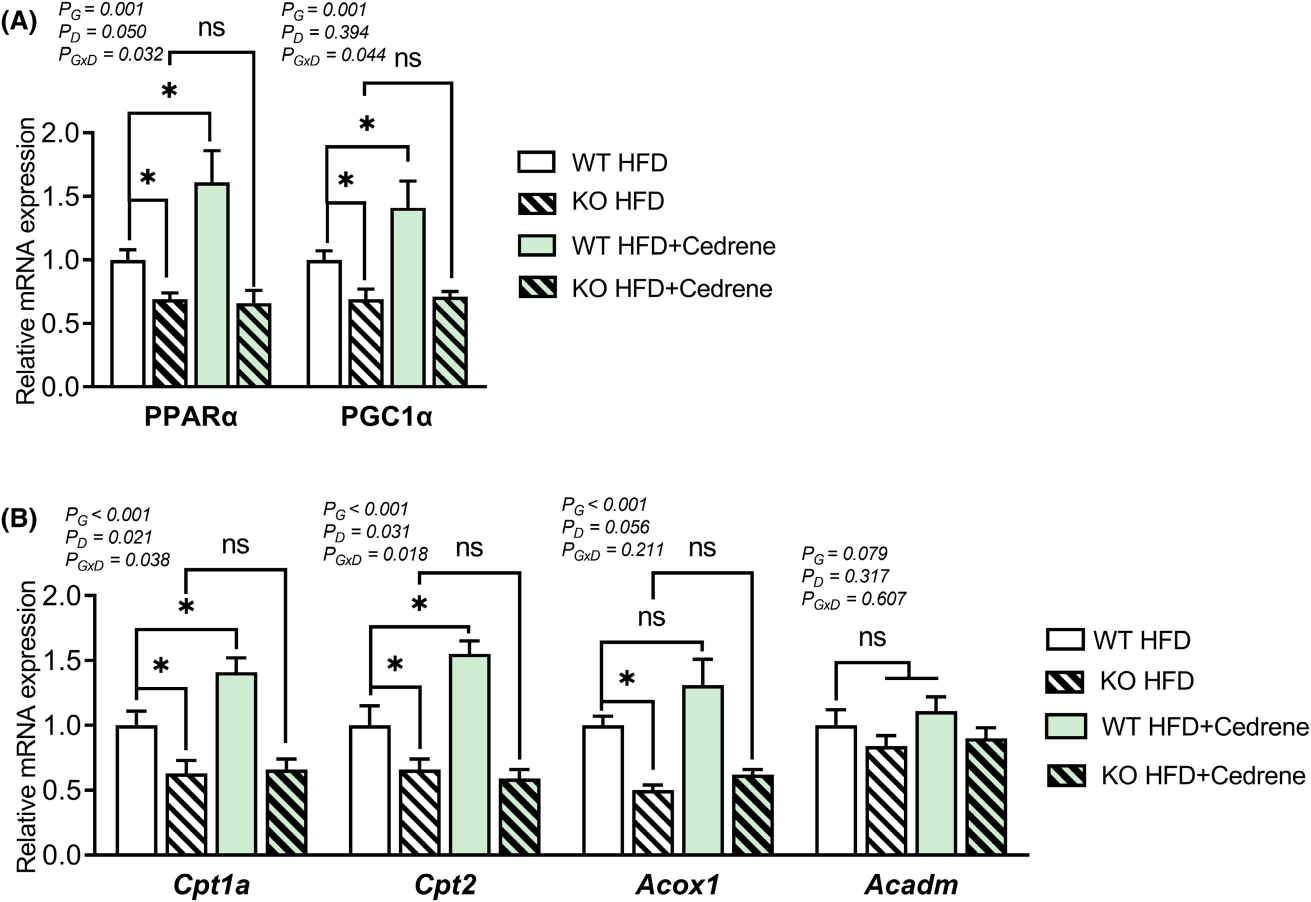
MOR23 regulates PPARα/PGC1α pathway and β‐oxidation genes in HFD‐fed mice. (A) mRNA expression of PPARα and PGC1α in the liver of MOR23 KO and WT mice fed an HFD, with or without cedrene treatment. (B) mRNA expression of carnitine palmitoyltransferase 1A (*Cpt1a*), *Cpt2*, acyl‐CoA oxidase 1 (*Acox1*), and acyl‐Coenzyme A dehydrogenase, medium chain (*Acadm*) in the liver of MOR23 KO and WT mice subjected to HFD, with or without cedrene treatment. Data are presented as means ± SEM. Samples from each group of eight mice were pooled into three biological replicates, with each replicate consisting of three, three, and two mice, respectively. A two‐way ANOVA was performed to assess the main effects (G, Genotype; D, Diet) and their interaction (G × D), as indicated within the graphs. Tukey's multiple‐comparison test was used to compare individual means, and statistical significance between groups is indicated by asterisks (*p < .05; ns, not significant).

Next, we evaluated several downstream mediators of AMPK, namely, peroxisome proliferator‐activated receptor α (PPARα) and peroxisome proliferator‐activated receptor‐γ coactivator 1‐α (PGC1α), which are known to activate the β‐oxidation pathway for fatty acids. Our findings demonstrated a significant decrease in the expression of both genes in KO mice fed an HFD compared to WT mice fed an HFD. Furthermore, the administration of the MOR23 ligand increased the mRNA expression of PPARα and PGC1α in HFD‐WT mice; however, this effect was not observed in HFD‐KO mice (Figure 6A).

Based on these observations, we hypothesized that upregulating the expression of PPARα and PGC1α would result in increased expression of β‐oxidation genes and enhanced β‐oxidation in the liver. To test this hypothesis, we examined the expression of key β‐oxidation genes including carnitine palmitoyltransferase 1A (*Cpt1a*), *Cpt2*, acyl‐CoA oxidase 1 (*Acox1*), and acyl‐Coenzyme A dehydrogenase, medium chain (*Acadm*). Our results revealed a significant decrease in the mRNA expression of *Cpt1a*, *Cpt2*, and *Acox1* in HFD‐KO mice compared to HFD‐WT mice. Additionally, the administration of cedrene significantly upregulated the expression of Cpt1a and Cpt2 in HFD‐WT mice; however, its effect on Acox1 was less pronounced, showing an increasing trend that did not reach statistical significance. The effects of cedrene were nullified in HFD‐KO mice. Although the effect on *Acadm* expression by MOR23 was observed, it did not reach statistical significance (Figure 6B). Together, these findings suggest that MOR23 plays a role in regulating the PPARα/PGC1α pathway and several β‐oxidation genes, potentially contributing to alterations in liver fat accumulation.

### 3.7 MOR23 plays a role in regulating lipogenesis and FFA uptake in the liver of mice under HFD

**FIGURE 7:**
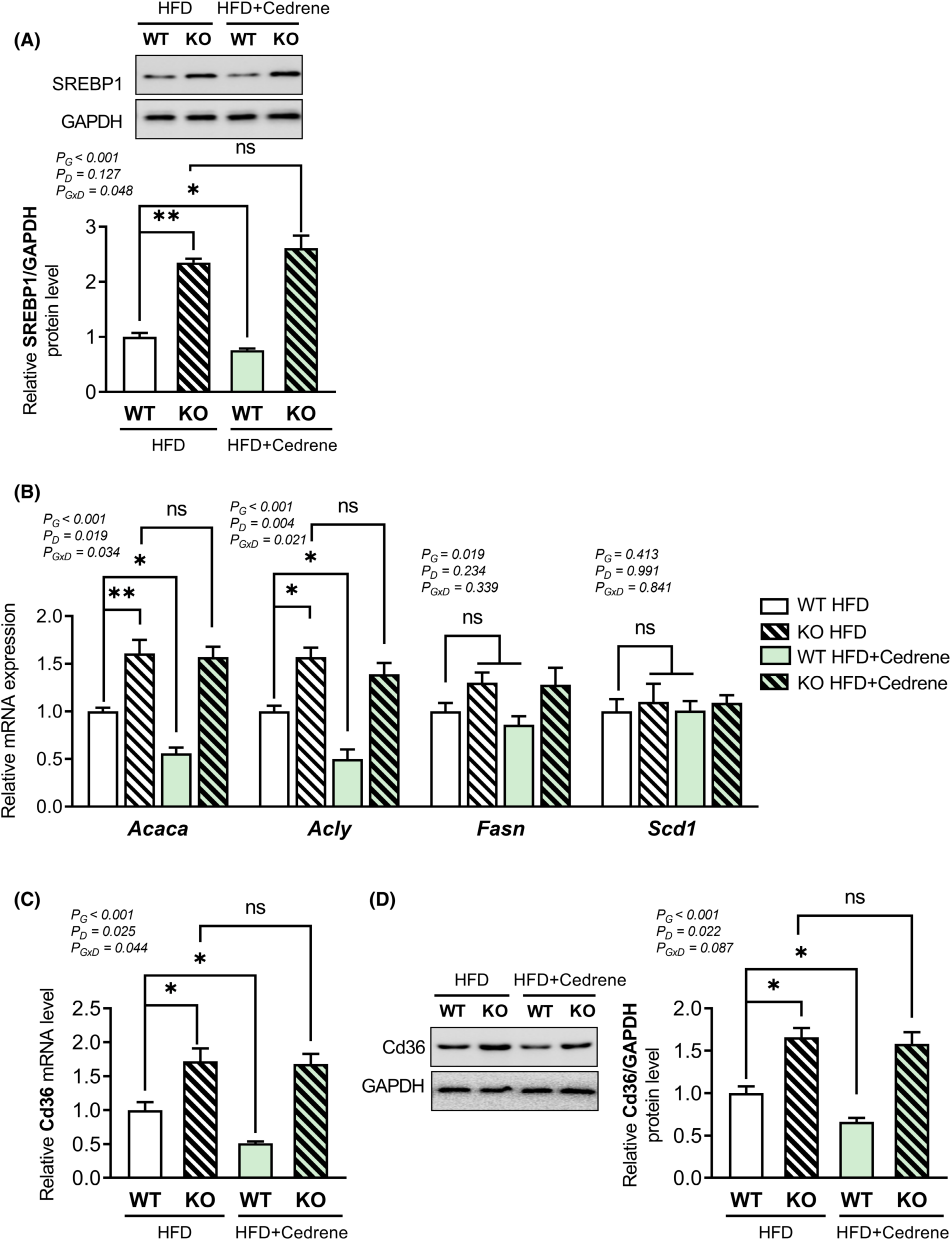
MOR23 plays a role in regulating lipogenesis and FFA uptake in the liver of mice under HFD. (A) Protein expression of SREBP1 in the liver of MOR23 KO and WT mice under HFD, with and without cedrene treatment. (B) mRNA expression of lipogenic genes acetyl‐Coenzyme A carboxylase alpha (*Acaca*), ATP citrate lyase (*Acly*), Fatty acid synthase (*Fasn*), and stearoyl‐Coenzyme A desaturase 1 (*Scd1*) in the liver of MOR23 KO and WT mice subjected to HFD, with and without cedrene treatment. (C) mRNA expression of FFA uptake transporter *Cd36* in the liver of MOR23 KO and WT mice under HFD, with and without cedrene treatment. (D) Protein expression of Cd36 (Cd36) in the liver of MOR23 KO and WT mice under HFD, with and without cedrene treatment. Data are presented as means ± SEM. Samples from each group of eight mice were pooled into three biological replicates, with each replicate consisting of three, three, and two mice, respectively. A two‐way ANOVA was performed to assess the main effects (G, Genotype; D, Diet) and their interaction (G × D), as indicated within the graphs. Tukey's multiple‐comparison test was used to compare individual means, and statistical significance between groups is indicated by asterisks (*p < .05; **p < .01; ns, not significant).

The activation of AMPK not only stimulates β‐oxidation but also inhibits lipogenesis and FFA uptake, leading to a potential reduction in liver fat accumulation. To explore the involvement of MOR23 in these pathways, the expression of SREBP1, a critical transcription factor in liver lipogenesis, was analyzed. Our results revealed a significant increase in SREBP1 protein expression in the livers of MOR23 KO mice. In contrast, treatment with cedrene significantly decreased SREBP1 protein levels in the livers of WT mice but did not affect SREBP1 levels in the livers of KO mice (Figure 7A).

It is widely accepted that hepatic SREBP1 increases the transcription of genes involved in de novo lipogenesis, such as acetyl‐Coenzyme A carboxylase alpha (*Acaca*), ATP citrate lyase (*Acly*), fatty acid synthase (*Fasn*), stearoyl‐coenzyme A desaturase 1 (*Scd1*), as well as the FFA uptake transporter *Cd36*.^24^, ^25^, ^26^ To determine whether MOR23 regulates one or both of these pathways downstream of SREBP1, the mRNA expression levels of lipogenic genes were measured following the knockout or activation of MOR23. As depicted in Figure 7B, the mRNA expression of Acaca and Acly was significantly increased in MOR23 KO mice compared to WT mice. In HFD‐fed WT mice, the expression levels of these genes were significantly decreased by MOR23 ligand consumption, which was not observed in HFD‐fed KO mice. The expression of Fasn showed an increasing trend in KO mice and appeared to decrease with the activation of MOR23 through cedrene administration, although these changes did not reach statistical significance. Meanwhile, Scd1 did not exhibit significant changes in response to either knockout or cedrene treatment.

Furthermore, the mRNA level of *Cd36* in the liver was analyzed and found to be significantly higher in KO mice than in WT mice. The expression level of *Cd36* was significantly reduced by cedrene in WT mice, while the reducing effect of cedrene was nullified in KO mice (Figure 7C). To investigate whether the changes in Cd36 mRNA expression correspond to changes at the protein level, we conducted a western blot analysis of hepatic Cd36 protein expression. Our results indicate that Cd36 protein levels were significantly higher in KO mice compared to WT mice. Furthermore, cedrene treatment significantly reduced Cd36 protein expression in WT mice; however, this reduction was not observed in KO mice (Figure 7D). Thus, MOR23 is thought to play a crucial role in regulating both lipogenesis and FFA uptake in the liver under HFD‐fed conditions.

## 4 DISCUSSION

Mice possess approximately 1000 olfactory receptor genes, while humans have around 400, with some overlap between the two species. While certain olfactory receptors are shared across species, either in a one‐to‐one or multiple‐to‐one correspondence, others remain unique to each.^27^, ^28^ In studies utilizing mouse models to investigate human olfactory receptors, particularly when there is a specific interest in human applications, those with a one‐to‐one relationship can be especially valuable for their potential to provide direct translational insights. One such receptor is MOR23 in mice, which corresponds to OR10J5 in humans.^29^ Our previous study demonstrated the crucial role of OR10J5 in the regulation of lipid accumulation in human hepatocytes.^22^ Building on these findings, our study suggests that OR10J5 may hold therapeutic potential for regulating hepatic lipid metabolism in humans, thereby extending insights derived from the mouse model to human health applications.

Some olfactory receptors are also found in nonolfactory tissues, with varying levels of expression depending on the tissue type.^10^, ^11^, ^30^, ^31^ According to GTEx data,^32^ olfr16 has been found to have the highest expression level in the liver, suggesting a significant role in this tissue. However, it is also expressed in other tissues. In this study, global MOR23 mice were used, which did not allow for distinguishing between direct hepatic and extrahepatic effects. Therefore, it is possible that systemic changes induced by MOR23 activation or knockout in other tissues could indirectly impact the improvement or worsening of hepatic steatosis. For instance, the activation of olfr78 in the kidney has been shown to mediate the secretion of several molecules that can have a chronic hypertensive effect on the complex array of pathways involved in blood pressure regulation.^33^ In the future, it will be important to confirm the expression of MOR23‐related pathologies in other tissues in MOR23 mice and to determine the role of hepatic MOR23 using a tissue‐specific mouse model.

Several G‐protein‐coupled receptors (GPCRs) have been reported to regulate fatty liver disease in in vivo.^34^ For instance, GPR55‐deficient mice show defective insulin signaling and a significant increase in liver fatty acid synthase, leading to hepatic steatosis.^35^ Additionally, GPR132 has been associated with hepatic lipid metabolism and gallstone development in mice, as GPR132‐deficient mice exhibit gallstones and an increased cholesterol saturation index.^36^ Despite these findings, practical applications for fatty liver therapy have not been developed due to the lack of knowledge about the ligand that activates the receptor (orphan receptor). In the case of MOR23, a specific GPCR, previous in vitro studies have identified cedrene as a potential ligand.^22^ This study further explores the protective role of cedrene through this receptor in an in vivo setting. Furthermore, a preclinical safety investigation of α‐cedrene showed that the no‐observed‐adverse‐effect level in rats was as high as 2000 mg/kg body weight per day (Kwang Dong Pharmaceutical Company, unpublished data). Additionally, α‐cedrene pharmacokinetic plasma levels increased linearly and showed rapid clearance; the oral bioavailability of α‐cedrene was relatively high (84.8%).^37^ Therefore, these findings suggest the possibility of developing cedrene as a medication or functional food targeting olfr16.

Hepatic steatosis is a complex disease with diverse genetic underpinnings that have been elucidated through gene‐KO studies, revealing two distinct patterns. The first pattern involves actively promoting lipid accumulation, even under normal dietary conditions, in mice lacking specific genes. These genes appear to act as principal switches governing the development of hepatic steatosis.^38^, ^39^, ^40^ In the second pattern, the genes do not play a role under normal conditions but become relevant when metabolic disorders such as HFD‐induced obesity occur.^41^, ^42^, ^43^ NAFLD is largely diet‐induced, and identifying potential treatment targets for these genes within this context represents a significant research avenue for controlling this disease. In the present study, we observed that HFD‐fed mice developed NAFLD within 10 weeks of feeding and that diet‐induced liver steatosis was significantly accelerated by the deletion of MOR23. Notably, the ND‐fed mice did not experience KO‐induced liver steatosis. These data suggest a functional role for MOR23 in lipid metabolism and/or hepatic distribution under metabolic stress conditions.

The three key pathways involved in hepatic lipid metabolism have been identified as β‐oxidation, lipogenesis, and fatty acid uptake. SREBP1, acting as a transcription factor in lipogenesis, plays a crucial role in promoting the expression of major lipogenic genes such as *Acaca*, *Acly*, *Fasn*, and *Scd1*. Additionally, SREBP1 has been reported to contribute to the increased expression of Cd36, a fatty acid transporter that enhances FFA uptake in the liver.^44^, ^45^, ^46^ In contrast, the transcription factors PPARα and PGC1α promote β‐oxidation by inducing the expression of *Cpt1a*, *Cpt2*, *Acox1*, and *Acadm*.^47^, ^48^, ^49^ Dysregulation of these pathways in hepatic steatosis results in excess lipid synthesis and decreased lipid degradation, leading to the accumulation of TG in the liver. Notably, it has been reported that these transcription factors selectively regulate hepatic lipid metabolism‐related target genes rather than employing an all‐or‐none system.^40^, ^43^, ^50^ In the present study, we found that the deletion of MOR23 greatly upregulated PPARα and PGC1α, along with the modulation of their target genes, including *Cpt1a*, *Cpt2*, and *Acox1*. Moreover, MOR23 deletion activated hepatic SREBP1 protein, leading to the upregulation of *Acaca*, *Acly*, and *Cd36*. Collectively, our results strongly suggest that deficiency in MOR23 can exacerbate the severity of hepatic steatosis, involving the modulation of multiple pathways.

A notable limitation of our study is the lack of precise cellular localization of MOR23 within liver tissue. Our previous results clearly demonstrate the functional role of OR10J5 (the human ortholog of MOR23) in hepatocytes, which constitute approximately 80% of the cell population of the liver.^22^ Furthermore, the Human Atlas single‐cell study reveals that OR10J5 is expressed in hepatocytes at a detectable transcript per million (TPM) level. In contrast, its expression is completely absent (0.0 TPM) in all other liver cell types, such as T cells, Kupffer cells, plasma cells, endothelial cells, and smooth muscle cells.^51^ Based on these findings, we speculate that MOR23 exerts its functional role possibly through hepatocytes in the liver. High‐resolution mapping would provide a more comprehensive understanding of its specific interactions within the liver's complex cellular environment. Given the scarcity of commercially available olfactory receptor antibodies, techniques such as RNAscope in situ hybridization could be employed in future studies to visualize the location of MOR23. Future research efforts should incorporate RNAscope or similar localization methods to further explore MOR23's role in liver biology.

Overall, this study reveals that MOR23 deficiency exacerbates hepatic steatosis in mice, particularly under a high‐fat diet, by disrupting lipid metabolism pathways including lipogenesis, fatty acid uptake, and β‐oxidation. The activation of MOR23 via its ligand cedrene mitigates these effects, highlighting the therapeutic potential of targeting MOR23 for nonalcoholic fatty liver disease. These findings provide a foundation for novel treatments that leverage olfactory receptor pathways to manage liver fat accumulation and related metabolic disorders.

## AUTHOR CONTRIBUTIONS

Wesuk Kang: Writing – Original Draft, Formal analysis, Data Curation, Investigation, Conceptualization. Suhjin Yang: Writing – Original Draft, Formal analysis. Jiyun Roh – Conceptualization. Dabin Choi – Conceptualization. Han‐Woong Lee – Resources. Jae Hoon Lee – Resources. Taesun Park – Conceptualization, Supervision, Project administration, Funding acquisition.

## DISCLOSURES

The authors declare no conflicts of interest.

## Supporting information


**Figure S1.**.

**Table S1.**.

## Data Availability

Data will be made available on request.
