# Supplementary material for: MOR23 deficiency exacerbates hepatic steatosis in mice
Source: FASEB J. 2024 Oct 17;38(20):e70107. doi: 10.1096/fj.202401468RR (PMC11580716; doi:10.1096/fj.202401468RR)
Supplement: Supplementary file 1 — Figure S1.. [file FSB2-38-e70107-s001.docx]

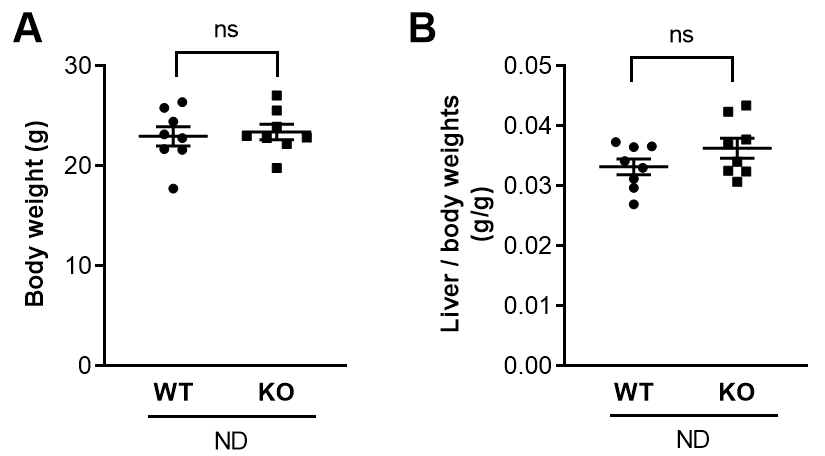


Supplementary Figure 1. (A) Examination of body weight in wild-type (WT) and MOR23 knockout (KO) female mice maintained on a normal diet (ND) for 10 weeks. (B) Assessment of liver weight relative to body weight in WT and MOR23 KO female mice. Data are represented as the mean ± standard error of the mean (SEM). n = 8 mice per group. “ns” denotes for not significant.


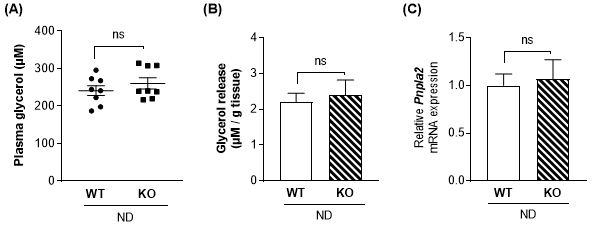


Supplementary Figure 2. (A) Plasma glycerol levels in WT and MOR23 KO mice fed ND. (B) Glycerol release from adipose tissue in WT and MOR23 KO mice. (C) Expression of lipolysis genes, specifically atglpatatin-like phospholipase domain-containing protein 2 (Pnpla2), in adipose tissue of WT and MOR23 KO mice. For (A), n = 8 mice per group. For (B), (C), samples from each group of eight mice were pooled into three biological replicates, with each replicate consisting of three, three, and two mice, respectively. No statistically significant (ns) difference was observed between the two groups.


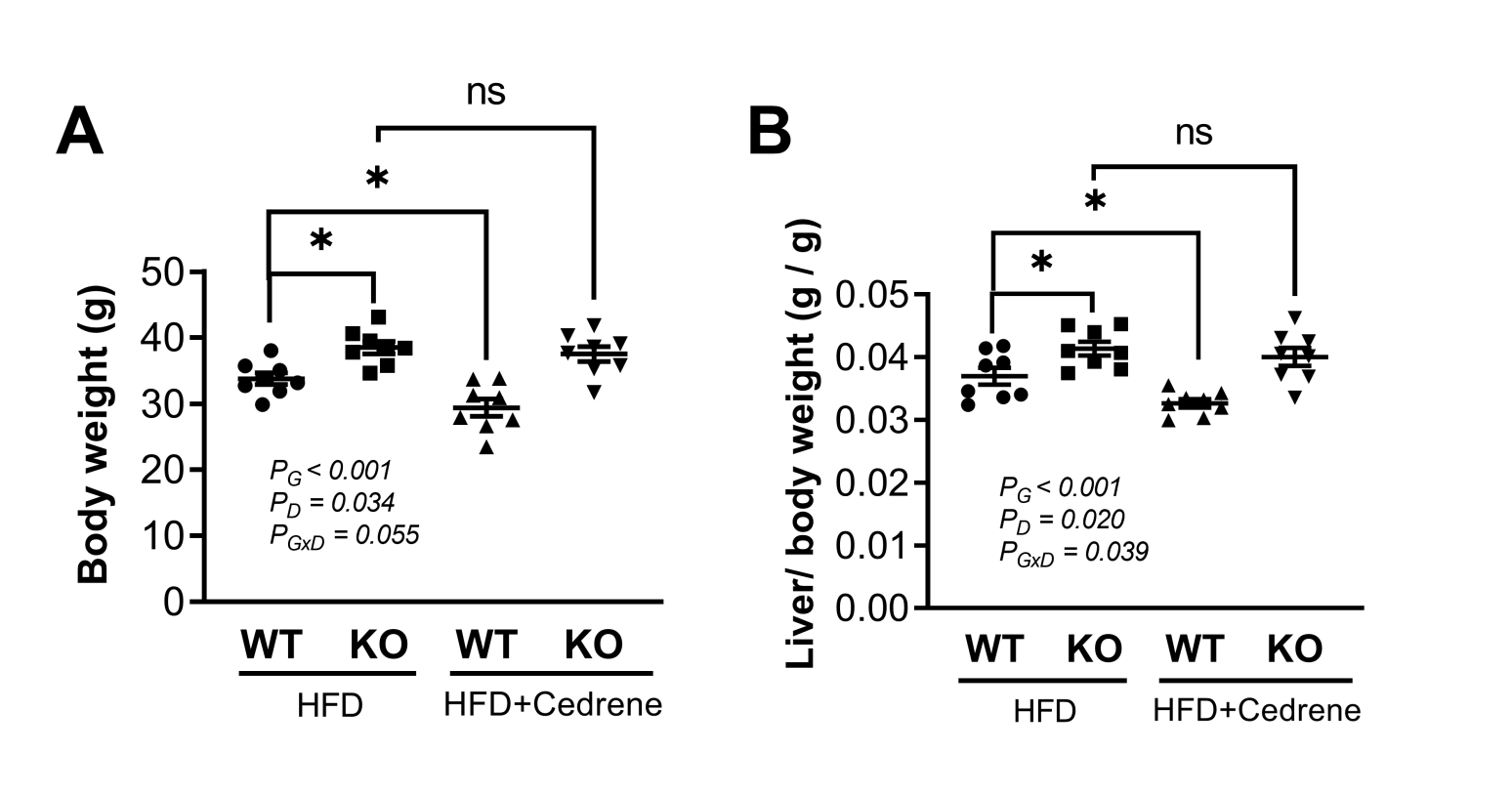


Supplementary Figure 3. (A) Body weight of WT and MOR23 KO female mice fed an HFD with or without cedrene treatment. (B) Liver weight normalized to body weight in WT and MOR23 KO female mice on an HFD with or without cedrene treatment. n = 8 mice per group. A two-way ANOVA was performed to assess main effects (G, Genotype; D, Diet) and their interaction (G×D), as indicated within the graphs. Tukey's multiple-comparison test was used to compare individual means, and statistical significance between groups is indicated by asterisks; ns, not significant; *p < 0.05.


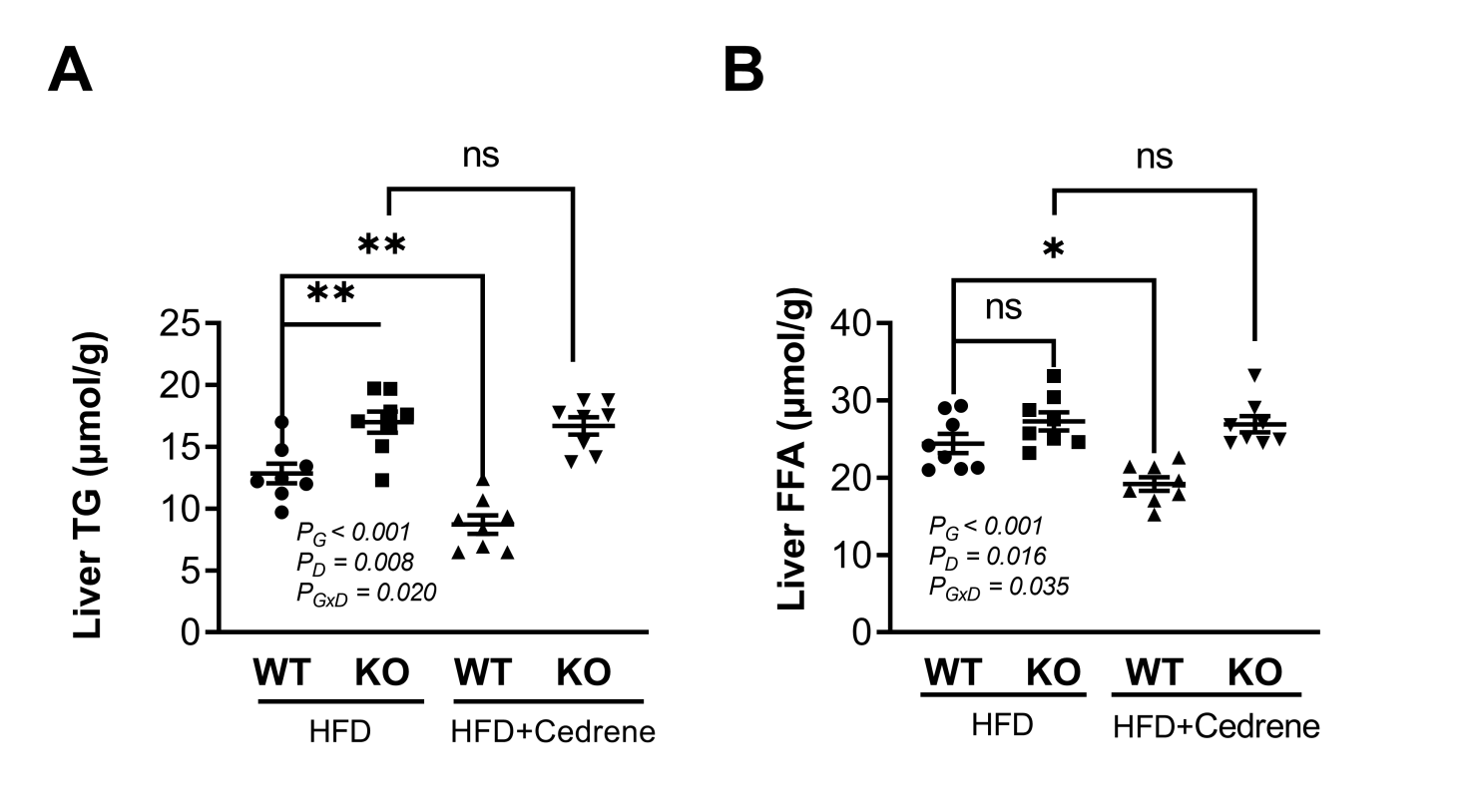


Supplementary Figure 4. Biochemical analysis of hepatic (A) triglyceride (TG) and (B) free fatty acids (FFA) levels in MOR23 KO and WT male mice fed an HFD and cedrene. The data are presented as the mean ± SEM (n = 8). A two-way ANOVA was performed to assess main effects (G, Genotype; D, Diet) and their interaction (G×D), as indicated within the graphs. Tukey's multiple-comparison test was used to compare individual means, and statistical significance between groups is indicated by asterisks; *p < 0.05; **p < 0.01; ns, not significant.
