# Supplementary material for: MOR23 deficiency exacerbates hepatic steatosis in mice
Source: FASEB J. 2024 Oct 17;38(20):e70107. doi: 10.1096/fj.202401468RR (PMC11580716; doi:10.1096/fj.202401468RR)
Supplement: Supplementary file 2 — Table S1.. [file FSB2-38-e70107-s002.docx]

**Supplementary Table 1. Composition of Experimental Diets: Normal diet (ND) and High-Fat Diet (HFD).**

| **Ingredient** | **ND**  **(g/kg diet)** | **HFD**  **(g/kg diet)** |
| --- | --- | --- |
| Casein^‡^ | 200.00 | 200.00 |
| D,L-methionine^†^ | 3.00 | 3.00 |
| Corn starch^‡^ | 150.00 | 111.00 |
| Sucrose^‡^ | 500.00 | 370.00 |
| Cellulose^‡^ | 50.00 | 50.00 |
| Corn oil^§^ | 50.00 | 30.00 |
| Lard^§^ | — | 170.00 |
| Mineral mixture (AIN-76)^§^ | 35.00 | 42.00 |
| Vitamin mix (AIN-76)^§^ | 10.00 | 12.00 |
| Choline bitartrate^†^ | 2.00 | 2.00 |
| Cholesterol^†^ | — | 10.00 |
| tert-Butylhydroquinone^†^ | 0.01 | 0.04 |
| **Total (g)** | **1,000** | **1,000** |

† Reagents were sourced from Sigma-Aldrich (St. Louis, MO, USA).
‡ Reagents were obtained from Duksan Pure Chemicals (Ansan, Korea).
§ Reagents were purchased from MP Biomedicals (Santa Ana, CA, USA).

**Supplementary Table 2.** Primer sequences for PCR

| Description | Sequence (5’→3′) |
| --- | --- |
| Acetyl-Coenzyme A carboxylase alpha (Acaca) | F: GCGCTATGGAAGTCGGCTATG  R: AGTTGTTGTCAGGCGAATGTTG |
| ATP citrate lyase (Acly) | F: ACTTGGGCCGGAACAAAAGC  R: TTTGCCGGTCTGCTCTGAAA |
| Acyl-Coenzyme A oxidase 1, palmitoyl (Acox1) | F: GAAGCCTGACGGCACGTA  R: TTCAGACTGGCGCCTCAC |
| Patatin-like phospholipase domain-containing protein 2 (Pnpla2) | F: CCAAGGGGTGCGCTATGT  R: GACGCGAAGCTCGTGGAT |
| cluster of differentiation 36 (Cd36) | F: TTAGTAGAACCGGGCCACGT  R: CACAGTTCCGATCACAGCCC |
| Carnitine palmitoyltransferase 1a (Cpt1a) | F: AGGTATGGCCACTTTGGGAC  R: GCTGGTCTTGCTGTGCATCT |
| Carnitine palmitoyltransferase 2 (Cpt2) | F: CAGGACCCTGCATACCAGC  R: GGGCATTGCGTCCTGAGTA |
| Fatty acid synthase (Fasn) | F: CAGCCAGGAGAATCGCAGTA  R: CTGCGATGAAGAGCATGGTT |
| Glyceraldehyde-3-phosphate dehydrogenase (Gapdh) | F: CTGGAAAGCTGTGGCGTGATG  R: AGGCCATGCCAGTGAGCTTC |
| Acyl-Coenzyme A dehydrogenase medium chain (Acadm) | F: CCGTCCGTTTTGCTTGCTG  R: GAGCCGCTTTCGAGTGTTG |
| Mouse olfactory receptor 23 (MOR23) | F: AGGAGGGGCGTT TTATTCACC  R: GAGATCCAAACATTAGAGCCCA |
| Peroxisome proliferator-activated receptor-γ coactivator 1-α (PGC1α) | F: TAAATCTGCGGGATGATGGA  R: GTTTCGTTCGACCTGCGTAA |
| Peroxisome proliferator-activated receptor α (PPARα) | F: TGACATGGAGACCTTGTGTATGG  R: AATCGGACCTCTGCCTCTTTG |
| Stearoyl-Coenzyme A desaturase 1 (Scd1) | F: TGGTGTCCAGCTTTGTTTCTTC  R: CAGCGGGGACTTGCTCTATC |
